# Does physical activity influence health behavior, mental health, and psychological resilience under the moderating role of quality of life?

**DOI:** 10.3389/fpsyg.2024.1349880

**Published:** 2024-03-11

**Authors:** Ru Liu, Rashid Menhas, Zulkaif Ahmed Saqib

**Affiliations:** ^1^College of Physical Education, Hunan City University, Yiyang, China; ^2^International Institutes of Medicine, The Fourth Affiliated Hospital Zhejiang University School of Medicine, Yiwu, Zhejiang, China; ^3^College of Urban Transportation and Logistics, Shenzhen Technology University, Shenzhen, China

**Keywords:** exercise, health functioning, structural equation modeling, enhancement, relationship

## Abstract

**Background:**

Physical activity significantly influences health-related behaviors, encompassing physical and mental well-being. Physical activity has been linked to enhancing health behavior, mental health, and psychological resilience. The current study is based on participants who were active in physical activity to improve health and well-being.

**Objectives:**

To examine the influences of physical activity on health behavior, mental health, and psychological resilience, considering the moderating role of quality of life.

**Method:**

A thorough cross-sectional online survey was conducted from April 15, 2023, to October 15, 2023. The survey was comprehensive and lasted for six months. The online poll received more than one thousand responses under convenience sampling. The selection criteria for the study were above 21 years old, and participants were active in physical activity to improve health and well-being. The collected data were analyzed using appropriate statistical SPSS-25 and SmartPLS 4.0 software to investigate the proposed research paradigm.

**Results:**

SEM results of model 1 (direct coefficients) show that PA has a positive effect on HeB, MeH, PsR, HeB on MeH, HeB on PsR. Out of six (in model 2), four moderating effects of QOL were significant, and two were statistically insignificant.

**Conclusion:**

It has been observed that the quality of life has a moderating role in the relationships between physical exercise and several aspects, such as psychological resilience, mental health, and health-related behavior. It is imperative to emphasize the importance of fostering consistent engagement in physical activity to cultivate a well-balanced and health-conscious way of life.

## 1 Introduction

Participating in consistent physical activity is crucial for maintaining optimal physical and mental health. Regular engagement in rigorous physical activity is necessary. Numerous empirical investigations have indicated that constant physical activity substantially impacts psychological resilience, mental well-being, and behavioral patterns, among other health-related attributes. Marashi et al. (2021) emphasized the significance of psychological assistance in addressing obstacles to physical activity during challenging circumstances, such as the COVID-19 epidemic. Additionally, they discussed the potential positive impact of exercise on mental well-being. Inquiry has time-honored constructive correspondence amid regular arrangement in physical activity and enhanced mental well-being. Regular physical activity is multifaceted since it impacts various aspects of an individual’s health and overall well-being (Menhas et al., 2021). The association between exercise and beneficial health outcomes has captured the attention of scientists. In a study conducted by Guo et al., it was observed that there was a negative correlation between the prevalence of diabetes and higher levels of physical activity, even after accounting for the influence of air pollution. The study provides additional evidence to support the beneficial impact of regular physical activity on overall well-being (Herbert, 2022).

Evaluating regular exercise’s impact on physical well-being and emotional satisfaction is paramount. It is widely recognized that regular physical activity is crucial in preventing and managing chronic illnesses (Saqib et al., 2020). It has also been proved that those who participate in consistent physical activity experience enhancements in their mental health and overall well-being. The encouragement of physical activity has long been recognized as a fundamental component in managing mental health disorders (Coyle et al., 2021; Barrera et al., 2022). Less physical exercise and more sedentary behavior are linked to health problems like depression, anxiety, and tension. A link between resiliency, mental health, and physical exercise has been found in older people. Maintaining a physical activity routine might help people be more mentally resilient (Reyes-Molina et al., 2022; Wermelinger Ávila et al., 2022).

Multiple studies have demonstrated that participation in physical activity directly impacts mental health, enhancing individuals’ outlook and emotional state. Physical exercise has been found to have many beneficial effects on an individual’s mental well-being. Research has indicated that this activity has the potential to enhance subjective well-being, mitigate stress and negative emotions, and enhance cognitive abilities. Engagement in sports and physical activity is associated with evident psychological, social, and physiological advantages. Initiating involvement at an early age and sustaining an active lifestyle yield enduring benefits for physical and mental well-being (Dai and Menhas, 2020; Duncan et al., 2020; Sang et al., 2021; Wei and Liu, 2023).

The significant impact of habitual physical activity on psychological resilience, mental health, and health behavior is the imperative of providing enough support for such activity to promote overall well-being (Feller et al., 2021). Both social and psychological factors play a significant influence in forming health-related habits. The inclusion of psychological elements in wellness efforts holds particular significance. It has been shown that aerobic and strength training routines can improve mental health and brain function (Fan et al., 2023).

## 2 The rationale of the study

**Figure 1:**
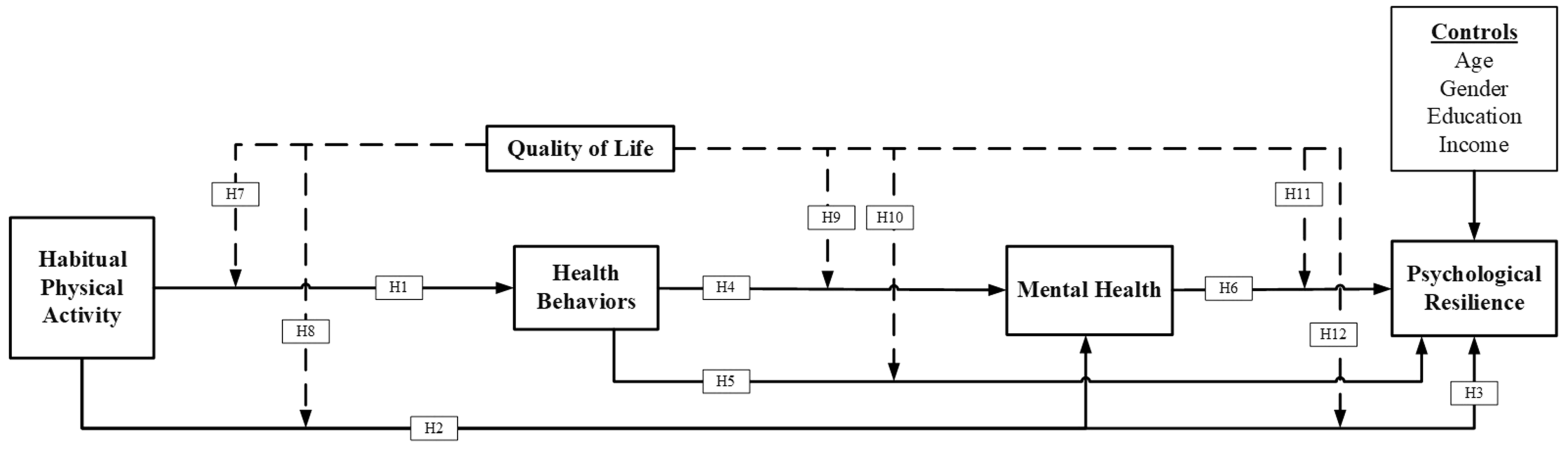
Conceptual framework.

Individuals across various age groups and with diverse physical capabilities might benefit significantly from consistent physical exercise. The significance of engaging in home-based exercise to prevent the decline of muscle mass associated with aging has been emphasized (Feller et al., 2021; Barrera et al., 2022). Physical activity significantly influenced health-related behaviors, encompassing physical and mental well-being, throughout diverse age cohorts and communities. Ensuring a harmonious equilibrium in an individual’s level of physical activity is therefore paramount for promoting mental well-being. Moreover, scholarly investigations exploring the correlation between physical activity and mental resilience have revealed that the latter positively impacts the former (Yang et al., 2022; Brito et al., 2023). It is often known that physical activity plays a significant role in enhancing overall well-being. In addition to improving physical health, physical activity also has a significant impact on psychological resilience and mental well-being. Despite the established advantages, there aren’t many studies investigating these relationships and considering quality of life as a moderating factor. For this reason, a deeper understanding of the complex relationships (See Figure 1) between these variables is needed. This study intends to close this gap by examining the influences of physical activity on health behavior, mental health, and psychological resilience, considering the moderating role of quality of life. The research indicates that regular physical activity has been associated with positive effects on the brain, including improvements in mood, cognitive performance, and the generation of markers related to synaptic plasticity and neurotrophic factors (Nowacka-Chmielewska et al., 2022). It has been emphasized that regular physical exercise in the first few years of life is crucial for healthy mental and physical development (Feller et al., 2021). Empirical research indicates that health behavior, mental health, and psychological resilience are significantly influenced by physical activity, psychological resilience, and quality of life, all of which are interrelated (Felipe et al., 2020; Sang et al., 2021). The current research has focused on the interaction between physical exercise, psychological resilience, mental health, and the moderating effect of quality of life. More evidence that the impact of physical activity on mental health may vary depending on activity level comes from a study that found a linear association between mental health variables and physical activity within a particular activity range (Shimura et al., 2023).

## 3 Hypothesis operationalization

### 3.1 Direct effects

Regular physical activity has been widely regarded as a compelling rationale for embracing a lifestyle that promotes overall well-being and substantially enhances one’s standard of living (Dai et al., 2021). There is a correlation between health behaviors and regular engagement in physical activity. The assertion is that health-related behaviors like regular physical activity tend to develop into habitual patterns over time due to repeated decision-making and implementation. Multiple research studies have substantiated the positive impacts of physical exercise on mental well-being (Dai and Menhas, 2020; Duncan et al., 2020; Saqib et al., 2020). Moreover, a positive correlation exists between increased levels of daily exercise, also known as habitual physical activity (HPA), and enhanced aerobic performance and an extended lifespan (Ma et al., 2021; Welsner et al., 2021). Consistent participation in physical activity yields enhancements in both bodily and mental well-being, with the adoption of additional health-enhancing practices and reduced susceptibility to chronic ailments (Saqib et al., 2020). The proposition that a correlation exists between consistent physical activity participation and heightened mental well-being levels has garnered significant attention in contemporary research. So, the given below hypotheses were developed;

*H1*: Physical activity positively associated with health behavior

*H2*: Physical activity positively associated with mental health

Physical activity in one’s free time is crucial to one’s mental and physical well-being. A consistent fitness regimen can contribute to the enhancement of an individual’s mental resilience. Teens with greater physical activity tend to have healthier mental states (Saqib et al., 2020). It has been shown that consistent participation in physical activity is linked to heightened resilience to stress in both mental and physical aspects (Saqib et al., 2020; Sang et al., 2021; Fan et al., 2023). Greater effects can be expected from strenuous physical activity (Dai and Menhas, 2020; Duncan et al., 2020; Saqib et al., 2020). Xu et al. (2021) discovered that exercise increased people’s resilience by satisfying fundamental psychological demands. Similarly, Lin et al. (2022) found a positive association between physical activity and self-esteem, psychological resilience, and interpersonal flexibility indicators in Chinese adolescents. A substantial body of empirical research supports the proposition that consistent participation in physical activity is positively associated with psychological resilience. So, the given below hypotheses were developed;

*H3*: Physical activity positively associated with psychological resilience

*H4*: Health behavior positively associated with mental health

Healthy behaviors can positively impact an individual’s emotional well-being (Dai and Menhas, 2020; Duncan et al., 2020; Saqib et al., 2020). Multiple investigations have provided evidence in support of this concept. An exemplary cross-sectional study was undertaken in the United Kingdom following the implementation of social distancing measures throughout the COVID-19 pandemic. In addition, Jacob et al. (2020) discovered a positive association between regular physical exercise and enhanced psychological well-being among participants from the general community in the United Kingdom. The study found a correlation between changes in physical activity levels among Danish university students during the COVID-19 pandemic and their mental health, indicating that maintaining a consistent physical activity routine is essential for promoting mental well-being (Petersen et al., 2023). Furthermore, research has indicated that students who engage in health-related programs demonstrate a higher propensity to pursue knowledge on mental health actively (Bożek et al., 2020). One plausible reason for this correlation is the provision of knowledge regarding the identification of symptoms associated with mental illness and the subsequent implementation of appropriate treatment strategies. So, the given below hypotheses were developed;

*H5*: Health behavior positively associated with psychological resilience

*H6*: Mental health positively associated with psychological resilience

### 3.2 The moderating effect of QOL

Studies have also examined the correlation between physical activity and quality of life in newborns, adolescents, and older adults regarding health-related quality of life (Moeini et al., 2021; Qin et al., 2021). The quality of life influences the relationship between physical activity and health behavior. Numerous research studies have supported that regular physical activity yields significant advantages for well-being and general life satisfaction (Dai and Menhas, 2020; Saqib et al., 2020; Fan et al., 2023). The empirical evidence supports that physical activity positively impacts physical and mental health and well-being (Dai and Menhas, 2020; Duncan et al., 2020). People also reported that being more active is linked to better general health, a higher standard of living, and fewer signs of depression and anxiety (Sang et al., 2021). Self-efficacy, general quality of life, and mental health were also positively related. Better mental health is linked to a higher quality of life (Bożek et al., 2020). Based upon the above arguments, it is assumed that “quality of life” positively moderates the relationship between physical activity, health behavior, and mental health. So, the given below hypotheses were developed;

*H7*: Quality of life positively moderates the relationship between physical activity and health behavior

*H8*: Quality of life positively moderates the relationship between physical activity and mental health

*H9*: Quality of life positively moderates the relationship between health behavior and mental health

Quality of life has a positive moderating effect on the association between physical activity and psychological resilience. The relationship between several factors and happiness is mediated by mental health (Fan et al., 2023). According to existing research, evidence supports that the “quality of life” factor has a constructive role in influencing the connection between regular physical activity and psychological resilience (Dai and Menhas, 2020; Saqib et al., 2020; Fan et al., 2023). Moreover, a research investigation carried out on individuals with physical disabilities revealed that the relationship between internalized stigma and psychological quality of life was mediated by resilience (Silván-Ferrero et al., 2020). Moreover, a research investigation conducted during the COVID-19 pandemic examined the impact of psychological resilience on the association between anxiety response, organizational commitment, and the quality of life among healthcare professionals (Son et al., 2022). The potential moderating effect of quality of life has also been investigated concerning the association between health-related quality of life and physical activity. The results of this study indicate a strong and persistent correlation between psychological resilience and quality of life, including many demographic characteristics and life conditions. There exists a body of evidence suggesting that engaging in exercise has a positive impact on health-related quality of life. However, additional research is required to ascertain the mechanisms and factors that moderate this relationship. So, the given below hypotheses were developed;

*H10*: Quality of life positively moderates the relationship between health behavior and psychological resilience

*H11*: Quality of life positively moderates the relationship between mental health and psychological resilience

*H12*: Quality of life positively moderates the relationship between physical activity, psychological resilience

## 4 Materials and methods

The study was carried out in China as part of the ongoing inquiry. The ethical principles outlined in the Helsinki Declaration of the World Medical Association were adhered to throughout the entirety of the research that was carried out for this particular study. The ethics committee of Hunan City University approved the study. Everyone who participated in the research project gave their informed consent after knowing the purpose of the study.

### 4.1 Study design and participants

A cross-sectional online survey was conducted from April 15, 2023, to October 15, 2023. The survey was comprehensive and lasted for six months. The online poll received more than one thousand responses under convenience sampling. After carefully performing the data quality check, a total of 743 responses (See Table 1) were selected for the data analysis (Faul et al., 2009). Further, the selection criteria for the study were above 21 years old, and participants were active in physical activity to improve health and well-being.

**Table 1:** Study participant’s demographic characteristics (N-743).

Variables	Categories	Frequency/Percentage
Gender	Male	387 (52.09%)
	Female	356 (47.91%)
Age	21–25	114 (15.34%)
	26–30	108 (14.54%)
	31–35	111 (14.94%)
	36–40	104 (14.00%)
	41–45	103 (13.86%)
	46–50	104 (14.00%)
	51+	99 (13.32%)
Education	High school graduate	169 (22.75%)
	College graduate	189 (25.44%)
	University graduate	198 (26.65%)
	Others	187 (25.17%)
Marital status	Single	201 (27.05%)
	Marred	191 (25.71%)
	Divorced	188 (25.30%)
	Widowed	163 (21.94%)
Occupation	Self-employed	237 (31.90%)
	Government Employee	248 (33.38%)
	Students	258 (34.72%)

The study’s participant demographics, comprising 743 individuals, reflect a balanced gender distribution, with 387 participants identifying as male (52.09%) and 356 as female (47.91%). Age distribution reveals a diverse representation across different age groups. Notably, participants aged 21–25 constitute 15.34%, followed by 26–30 (14.54%), 31–35 (14.94%), 36–40 (14.00%), 41–45 (13.86%), 46–50 (14.00%), and those aged 51 and above at 13.32%. Educational backgrounds vary among participants, with 22.75% having completed high school, 25.44% being college graduates, 26.65% holding university degrees, and 25.17% falling into the ‘Others’ category. Marital status is diverse, including 27.05% single individuals, 25.71% married, 25.30% divorced, and 21.94% widowed. In terms of occupation, participants are engaged as self-employed (31.90%), government employees (33.38%), and students (34.72%).

### 4.2 Survey measures

The researchers designed a self-administered questionnaire based on the previous study’s literature and data collection instruments to acquire the primary data. Before administering the final survey, a preliminary assessment (a pilot test) with a sample size of 25 participants was conducted to determine the response rate. Following the completion of the pilot study, the questions were subjected to a series of edits to achieve the maximum possible number of responses. The questionnaire included both closed-ended and open-ended questions, in addition to items based on a Likert scale. The survey measures are used to collect the data for the current study.

#### 4.2.1 Physical activity

Regular physical exercise affects mental health, health behavior, psychological resilience, and quality of life. The association between regular physical exercise and its impact on health behavior, mental health, and psychological resilience must be investigated further to understand the relationship between them better. The scale was adopted from the study (Saqib et al., 2020). A five-point Likert scale (almost every day, four to five days every week, three days per week, one to two days per week, and sometimes) was used to measure the physical activity of the study participants (walking outside for exercise, walking with a pet; engage in light activities such as bowling, billiards, golf with a cart, and shuffleboard; engage in moderate activities such as doubles tennis, dancing, hunting, and skating; engage in strenuous activities such as jogging, swimming, and cycling; and engaging in social activities such as book club, museum visiting, and green park visiting).

#### 4.2.2 Health behavior

Health behavior describes individuals’ activities and decisions concerning their health. The health-related practices of the elderly population in China have changed with time, influenced by many factors (Dai et al., 2021; Fan et al., 2023). Health behavior technology engagement can be divided into two distinct components, which collectively measure the effectiveness of digital interventions aimed at modifying behavior: health behavior engagement and behavior change intervention engagement (Cole-Lewis et al., 2019; Fang et al., 2022). The scale was adopted from the study (Fan et al., 2023). A five-point Likert scale (strongly disagree, disagree, neutral, agree, and strongly agree) was used to measure the study participant’s health behavior.

#### 4.2.3 Quality of life

Numerous research studies have established a correlation between exercise and life satisfaction (Saqib et al., 2020; Sang et al., 2021; Fan et al., 2023). The positive impacts of physical exercise on various health-related quality-of-life indices demonstrate that consistent participation can enhance the well-being and satisfaction of important subpopulations (Dai et al., 2021; Fan et al., 2023). A five-point Likert scale (poor, fair, good, very good, and excellent) was used to measure the quality of life of the study participants (mental health over the past four weeks, physical health over the past four weeks, psychiatric consultation, participation in social, community, and civic activities and overall well-being). Quality of life and health-related quality of life (HRQoL) are linked to exercise (Saqib et al., 2020).

#### 4.2.4 Mental health

The impact of consistent physical activity on a person’s mental health and psychological resilience is a topic that has garnered much attention from researchers working in health and psychology (Fan et al., 2023). Participating in physical activity has been shown in multiple studies to positively impact various aspects of health and well-being, which points to the importance of this connection (Saqib et al., 2020). The scale was adopted from the study (Fan et al., 2023). A five-point Likert scale (none of the time, rarely, some of the time, often, and all the time) was used to measure the study participant’s health behavior (feeling optimistic about the future, feeling useful, feeling relaxed, dealing with problems well, thinking clearly, feeling close to other people and able to make up my mind about things).

#### 4.2.5 Psychological resilience

The presence of psychological resilience as a moderator in the relationship between life satisfaction and the elderly sheds light on the significant role that quality of life plays as a mediator in the connection between regular physical exercise and its impact on mental well-being (Zheng et al., 2020). According to the concept of psychological resilience proposed by De Terte et al. (2014), participation in physical exercise is an essential component that should be included. A five-point Likert scale (completely disagree, disagree, neutral, agree, and completely agree) was used to measure the study participant’s health behavior (confidence in myself, ability to adjust to difficult situations, ability to preserve, easily pick up after setbacks, resilient, unexpected problems can cope, myself appreciate, same time can handle a lot and believe in myself).

### 4.3 Data analysis

Descriptive statistics were computed using the SPSS-25 software to outline the characteristics of the study sample, comprised of 743 individuals who participated in the research. The present investigation employed structural equation modeling (SEM) with the assistance of the SmartPLS 4.0 software to investigate the proposed research paradigm. It was decided to use the SEM technique, specifically the partial least squares (PLS) method, to analyze the structures and the indicators. For the aim of putting the model through its paces, the SEM methodology is put to use. Examining the factor loadings connected with each construct and determining whether or not discriminant and convergent validity are present are the steps that need to be taken to arrive at the usual values for variance extraction. As a consequence of this, a multivariate analytic approach is utilized to investigate the interrelationships that exist between the variables that comprise the conceptual model.

## 5 SEM analysis

### 5.1 Measurement reliability and validity

To verify the moderating effect of QOL, we prepared two different structural models (Model 1 = structural model excluded QOL’s effect; Model 2 = structural model included QOL’s effect). In measuring the proposed models, factor analysis was used to measure the construct’s reliability and validity through smartPLS 4.0 statistical software (Henseler et al., 2016). Before factor analysis, the standardized squared mean squared residual (SRMR), unweighted minimum squares discrepancy (d_ULS), geodetic discrepancy (d_G), Chi-square [X (Barrera et al., 2022)], and normed fit index (NFI) values were calculated to measure the fitness of M1 and M2. The calculated values of SRMR, d_ULS, d_G, [X (Barrera et al., 2022)], and NFI were acceptable and within the limit (See Table 2). Model fitness calculation provided saturated values, which imply the model acceptance for the structural equation modeling (Browne and Cudeck, 1993). To measure the reliability of constructs, Values for composite reliability (rho_a and rho_c), average variance extracted (AVE), and Cronbach’s alpha of each construct were calculated (See Table 3). In both models, the calculated values for CRs were more than the 0.7 threshold, AVEs were above the 0.50 threshold, and Cronbach’s alpha values were more than the 0.7 threshold (Carmines and Zeller, 1979). To measure the validity of constructs, we employed convergent and discriminant validity tests through AVEs. Convergent validity (CV) focuses on concept similarities, and discriminant validity (DV) focuses on differences (Cheung and Wang, 2017). AVEs index was used to assess CV, and calculated values were sufficient to confirm CV. Fornell–Larcker ratio and Heterotrait–Monotrait ratio of correlations (HTMT) standards were employed for DV confirmation. For the Fornell–Larcker ratio, the square root of AVEs was compared with inter-correlation values (See Table 4). All inter-correlations were lower than the square root of AVEs, which is sufficient for DV confirmation (Cheung and Wang, 2017). Further, variance inflation factor (VIF) values were examined for multicollinearity issues. The threshold for VIFs is <5.0, indicating that data have no multicollinearity problem.

**Table 2:** Model fitness for M1 and M2.

Fit indices	Structured model 1	Structured model 2
Standardized squared mean squared residual (SRMR)	0.061	0.061
Unweighted minimum squares discrepancy (d_ULS)	1.539	1.858
Geodetic discrepancy (d_G)	0.438	0.498
Chi-square	1743.356	1998.463
Normed fit index (NFI)	0.849	0.848

**Table 3:** Factor analysis.

Items	PA	HeB	MeH	PsR	QoL	VIF	Cronbach’s alpha	Composite reliability (rho_a)	Composite reliability (rho_c)	Average variance extracted (AVE)
PA1	**0.825**	0.537	0.444	0.488	−0.412	2.062	0.866	0.867	0.903	0.652
PA2	**0.803**	0.544	0.422	0.479	−0.418	1.878				
PA3	**0.788**	0.503	0.456	0.495	−0.390	1.780				
PA4	**0.794**	0.505	0.460	0.453	−0.410	1.841				
PA5	**0.825**	0.572	0.453	0.502	−0.410	2.030				
HeB1	0.528	**0.748**	0.531	0.548	−0.399	1.508	0.801	0.802	0.862	0.556
HeB2	0.470	**0.766**	0.512	0.569	−0.415	1.583				
HeB3	0.514	**0.745**	0.487	0.540	−0.351	1.508				
HeB4	0.480	**0.748**	0.485	0.526	−0.383	1.545				
HeB5	0.466	**0.721**	0.474	0.467	−0.356	1.478				
MeH1	0.470	0.529	**0.805**	0.495	−0.392	2.117	0.900	0.901	0.921	0.626
MeH2	0.441	0.509	**0.795**	0.454	−0.414	2.053				
MeH3	0.412	0.567	**0.780**	0.472	−0.370	1.990				
MeH4	0.445	0.554	**0.781**	0.469	−0.428	1.947				
MeH5	0.441	0.526	**0.798**	0.456	−0.428	2.084				
MeH6	0.439	0.522	**0.799**	0.464	−0.394	2.078				
MeH7	0.414	0.493	**0.777**	0.451	−0.378	1.945				
PsR1	0.630	0.746	0.622	**0.750**	−0.488	1.877	0.906	0.918	0.923	0.574
PsR2	0.468	0.531	0.462	**0.859**	−0.438	3.820				
PsR3	0.426	0.504	0.439	**0.832**	−0.416	3.493				
PsR4	0.461	0.530	0.463	**0.861**	−0.428	3.755				
PsR5	0.445	0.553	0.417	**0.790**	−0.379	2.618				
PsR6	0.252	0.278	0.263	**0.616**	−0.274	1.765				
PsR7	0.377	0.456	0.402	**0.741**	−0.381	2.460				
PsR8	0.406	0.435	0.396	**0.692**	−0.384	1.799				
PsR9	0.462	0.617	0.411	**0.636**	−0.366	1.498				
QoL1	−0.366	−0.381	−0.361	−0.413	**0.781**	1.715	0.812	0.811	0.869	0.570
QoL2	−0.382	−0.350	−0.361	−0.376	**0.760**	1.651				
QoL3	−0.359	−0.370	−0.419	−0.345	**0.765**	1.662				
QoL4	−0.420	−0.429	−0.390	−0.442	**0.727**	1.427				
QoL5	−0.374	−0.393	−0.379	−0.425	**0.741**	1.509				

Bold values are factor loadings; PA, Physical activity; HeB, Health behavior; MeH, Mental health; PsR, Psychological resilience; QoL, Quality of life.

**Table 4:** Discriminant validity.

Constructs	PA	HeB	MeH	PsR	QoL
*Heterotrait–Monotrait ratio (HTMT) – matrix*					
PA	**1.000**				
HeB	0.791	**1.000**			
MeH	0.627	0.787	**1.000**		
PsR	0.652	0.801	0.631	**1.000**	
QoL	0.601	0.631	0.592	0.607	**1.000**
*Fornell–Larcker criterion*					
PA	**0.807**				
HeB	0.660	**0.746**			
MeH	0.554	0.669	**0.791**		
PsR	0.599	0.712	0.589	**0.758**	
QoL	−0.505	−0.512	−0.507	−0.533	**0.755**
95% CI-upper limits	0.429	0.467	1.601	1.380	0.542
95% CI-lower limits	−0.208	−0.228	−0.881	−0.728	−0.285

The diagonal bold values are under the root of AVEs: PA, Physical activity; HeB, Health behavior; MeH, Mental health; PsR, Psychological resilience; QoL, Quality of life.

### 5.2 Hypotheses testing

**Figure 2:**
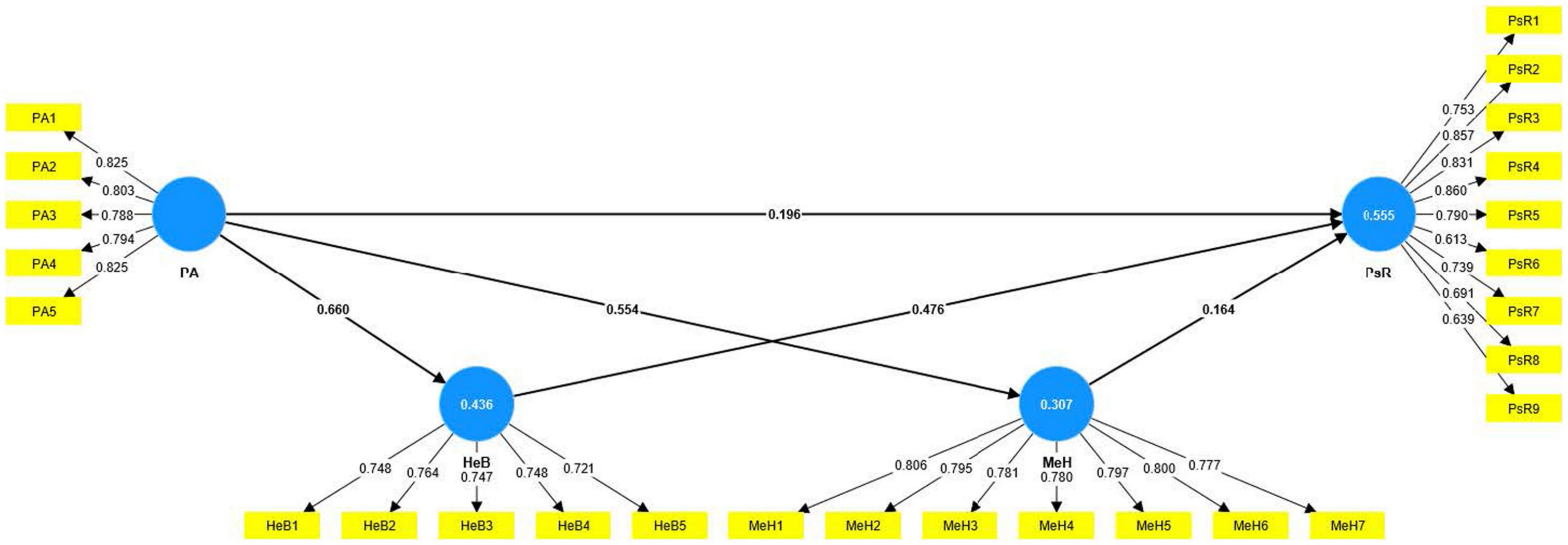
SEM results for structural model 1.

**Figure 3:**
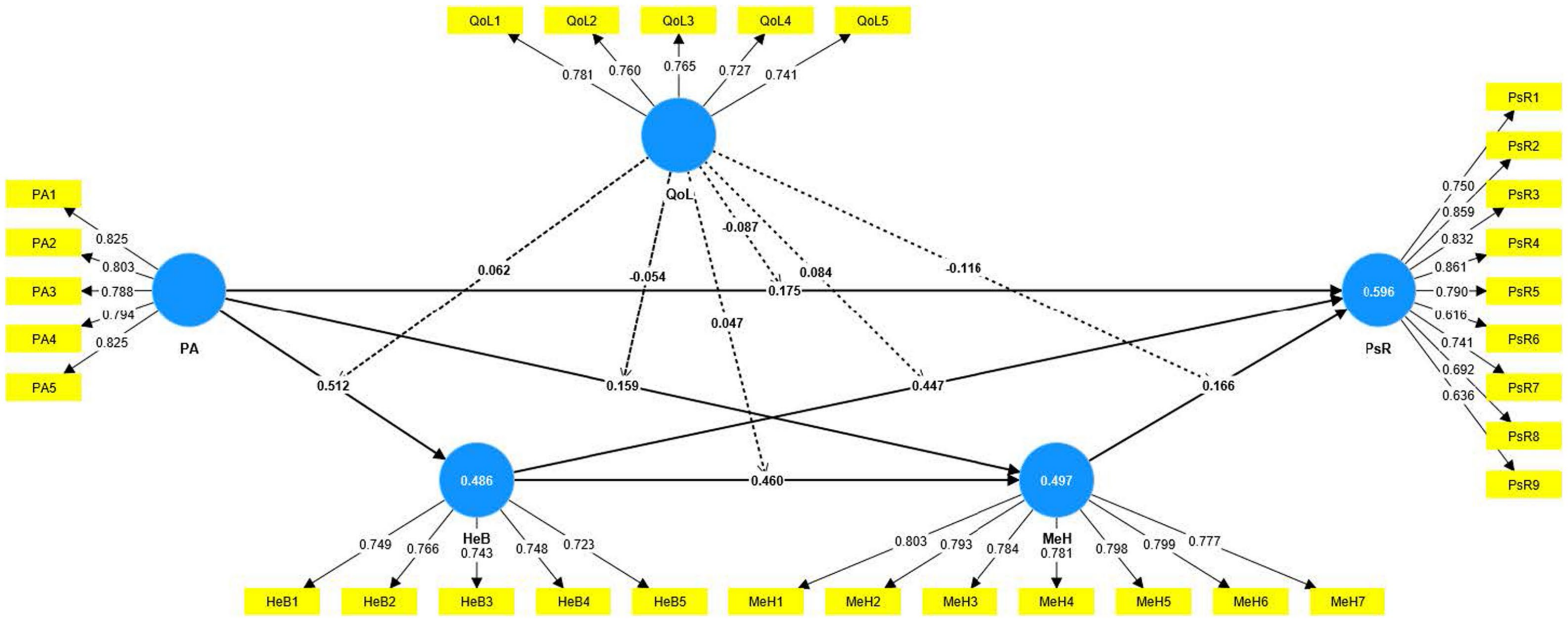
SEM results for structural model 2.

The structural equation modeling (SEM) technique examined the proposed hypotheses through the smartPLS 4.0 statistical package. Before running analyses, all constructs were standardized through factor analysis. In SEM analysis, path coefficients (β), standard deviation, *p*-values, and *T*-values were extracted to decide the acceptance or rejection of each hypothesis (Table 5). Six direct hypothetical paths, as well as six moderating paths, were tested. Where the path coefficient is the effect (change for every 1 unit) of the predictor on the outcome variable, the significance level is linked with the *p*-value. In structural model 1, six direct hypothetical paths were tested (See Figure 2), and the QOL concept was employed as a moderator (having six moderating hypothetical paths) in structural model 2 (See Figure 3 and Table 5).

**Table 5:** SEM results.

Path coefficients	Original sample (O)	Standard deviation (STDEV)	*T* statistics (|O/STDEV|)	*p*-values
*Structural model 1*				
PA → HeB	0.660	0.035	19.031	0.000
PA → MeH	0.554	0.040	4.967	0.000
PA → PsR	0.196	0.032	6.195	0.000
HeB → MeH	0.539	0.044	12.232	0.000
HeB → PsR	0.476	0.037	12.775	0.000
MeH → PsR	0.164	0.034	4.793	0.000
*Structural model 2*				
PA → HeB	0.512	0.045	11.472	0.000
PA → MeH	0.159	0.042	3.762	0.000
PA → PsR	0.175	0.033	5.237	0.000
HeB → MeH	0.460	0.047	9.861	0.000
HeB → PsR	0.447	0.039	11.347	0.000
MeH → PsR	0.166	0.039	4.262	0.000
QoL × HeB → MeH	0.047	0.030	1.579	0.114
QoL × HeB → PsR	0.084	0.041	2.041	0.041
QoL × PA → HeB	0.062	0.025	2.444	0.015
QoL × PA → MeH	−0.054	0.039	1.394	0.164
QoL × PA → PsR	−0.087	0.036	2.443	0.015
QoL × MeH → PsR	−0.116	0.049	2.336	0.020

PA, Physical activity; HeB, Health behavior; MeH, Mental health; PsR, Psychological resilience; QoL, Quality of life.

SEM results show that PA has a positive effect on HeB with (M1 = 0.660 β-value, 19.031 *t*-value, and *p* = < 0.000 and Model 2 = 0.512 β-value, 11.472 *t*-value, and *p* = < 0.000) which supported to hypothesis (H1). The results for H2 indicate that the proposed hypothesis confirms the positive effect of PA on MeH with (model 1 = 0.554 β-value, 4.967 *t*-value, and *p* = < 0.000 and Model 2 = 0.159 β-value, 3.762 *t*-value, and *p* = < 0.000). The calculated coefficients (β), *t*-value, and *p*-value [β = 0.196, T = 6.195, *p* = < 0.000 (model 1) and β = 0.175, T = 5.237, *p* = < 0.000 (Model 2)] for the effect of PA on PsR which show confirmation of hypothesis (H3). The effect of HeB on MeH (H4) was statistically supported with (Model 1 = 0.539 β-value, 12.232 *t*-value, and *p* = < 0.000 and Model 2 = 0.460 β-value, 9.861 *t*-value, and *p* = < 0.000). According to the SEM outcomes, HeB has a positive effect on PsR in the structural model 1 with β = 0.476, T = 12.775, *p* = < 0.000, and on PsR in the structural model 2 the β = 0.447, T = 11.347, *p* = < 0.000, that supported to hypothesis (H5). For hypothesis (H6), the SEM analysis claimed a significant positive effect of MeH on PsR with (Model 1 = 0.164 β-value, 4.793 *t*-value, and *p* = < 0.000 and Model 2 = 0.166 β-value, 4.262 *t*-value, and *p* = < 0.000).

According to SEM analysis, the six direct effects among PA, HeB MeH, and PsR are different in structural model 1 from model 2, which shows a moderating role of QOL. Out of six, four moderating effects of QOL were significant, and two were statistically insignificant. For example, the moderating effect of QOL on the relationship between PA and HeB significantly existed with β = 0.062, T = 2.444, and *p* = < 0.05, which confirms hypothesis (H7). The results for hypothesis (H8) and (H9) indicate the insignificance of moderating effects of QOL for PA➔MeH and HeB➔MeH with β = −0.054, T = 1.394, *p* = 0.164 as well as β = 0.047, T = 1.579, *p* = 0.114 values. The moderating effect of QOL on the relationship between HeB and PsR is significant and supported hypothesis (H10) with β = 0.084, T = 2.041, *p* = < 0.05 values. For hypothesis (H11), The calculated coefficients (β), *t*-value, and *p*-value are β = −0.116, T = 2.336, and *p* = < 0.05, showing the confirmation of the moderating effect of QOL on MeH➔PsR. Lastly, the moderating effect of QOL (H12) on the linkage between PA and PsR was significantly supported with β = −0.087, T = 2.443, *p* = < 0.05.

## 6 Discussion

Engaging in regular physical activity has a beneficial effect on health-related behaviors, mental well-being, and psychological resilience. Numerous studies have shown that frequent and strenuous physical activity positively affects a person’s health and well-being in general (Saqib et al., 2020; Sang et al., 2021; Fan et al., 2023). There is a correlation between regular physical activity and improvements in one’s mental well-being, psychological resilience, and total subjective well-being (Feil et al., 2021; Peng et al., 2022). Our study also found similar results that regular physical activity positively impacts mental well-being and psychological resilience. Regular physical activity is connected with good outcomes for health behavior, such as increased self-efficacy and positive affective appraisals. Regular physical activity as part of one’s routine and way of life might help strengthen one’s mental resilience. Psychological resilience can modify the effect of stress and anxiety on the overall quality of life (Liu et al., 2022). The habit formation hypothesis provides a useful framework for analyzing the impact of regular participation in physical activity on psychological well-being, psychological resilience, and behaviors that are beneficial to one’s health (Hagger, 2019). A significant link exists between regular physical activity and improved psychological well-being, psychological resilience, and positive health behaviors (Menhas et al., 2023).

Participating in regular and enough physical activity benefits an individual’s overall health. Physical activity can improve several facets of mental well-being (Liu et al., 2022; Menhas et al., 2023). Our findings are similar: physical activity benefits one’s general well-being and contentment with life. Resiliency can change the relationship between the consequences of one’s physical and mental health. In addition, a resilient mindset can reduce the risk of health problems and improve general well-being (Moore et al., 2015). Regular physical activity yields significant health benefits and mitigates the likelihood of chronic illness occurrence (Saqib et al., 2020). The correlation between regular physical activity and enhanced mental health, alongside its physiological advantages, has been shown in our findings. The association between consistent engagement in physical activity and several dimensions of quality of life, including psychological well-being, mental health, and overall quality of life, has been found. Similar results were reported by Park and Park (2022) that physical activity positively impacts psychological well-being, mental health, and overall state of being. Furthermore, a scholarly investigation by Lyons et al. (2020) examined the impact of COVID-19 on the psychological well-being and adaptive strategies employed by medical students in Australia. The results of this study underscore the significance of physical exercise in mitigating stress levels and promoting general well-being.

Promoting healthy behaviors such as seeking knowledge, participating in physical exercise, and adhering to a well-balanced diet to enhance mental well-being is vital for quality of life. The study conducted by Oftedal et al. (2019) revealed a positive correlation, like our findings, between better behavior patterns and many beneficial outcomes, including a decrease in psychological distress, an enhancement in self-rated health, and an overall improvement in quality of life, including both physical and mental well-being. Quality of life positively influences the association between physical activity and health behavior. Similarly, Almeida et al. (2022); Marconcin et al. (2022); and Leridis et al. (2023) offer evidence to support the concept that quality of life positively moderates the association between regular physical activity and mental health. It has been shown that “quality of life positively moderates the relationship between mental health and psychological resilience.” In addition, it is also indicated that psychological resilience plays a vital role in determining an individual’s overall quality of life (Mejia-Lancheros et al., 2021). There are numerous and intricate relationships between the effects of physical activity on psychological resilience, mental health, and health-promoting behaviors. Physical activity has been demonstrated to improve mental health, life satisfaction, and general health behaviors (Gothe et al., 2020).

Different demographics provide background for the impact of physical exercise on health behavior and psychological resilience (Fang et al., 2022). It is significant to highlight the intricate and nuanced relationship between physical exercise and health outcomes. Psychological resilience can be influenced by various factors, including the type and intensity of the activity as well as personal beliefs and habits (Cioffi et al., 2010). Recreational activity involvement has been demonstrated to favorably increase physical self-efficacy, positively affecting quality of life, suggesting a possible mechanism by which physical exercise improves quality of life (Leifa and Zheleznyak, 2017; Su-ul and Soon-il, 2018). Overall, the findings of our study are similar to the abovementioned arguments. It is essential to consider that this relationship is influenced by the quality of life experienced by the individual. Quality of life positively moderates the relationship between health behavior and mental health. These results show the importance of putting quality of life first to improve physical and mental health.

## 7 Conclusion

The relationship between physical exercise and health behavior, mental health, and psychological resilience is intricate and multidimensional, and quality of life plays a crucial moderating role in these associations. Frequent exercise benefits psychological resilience, mental health, and health behaviors. Based on the results of this study, it is imperative to emphasize the importance of fostering consistent engagement in physical activity to cultivate a well-balanced and health-conscious way of life. It has been observed that the quality of life has a moderating role in the relationships between physical exercise and several aspects, such as psychological resilience, mental health, and health-related behavior. The results emphasize the multifaceted influence of physical activity on several aspects of well-being, underscoring its importance in fostering comprehensive health. The specifics of these correlations and the potential moderating effects of quality of life need more research, even though physical exercise has been linked to several favorable outcomes, such as improved quality of life, mental health, and health behavior. The significance of advocating for and sustaining a physically active lifestyle to enhance overall health and quality of life is underscored by the positive association between consistent physical exercise and several aspects of health and well-being.

### 7.1 Limitations of the study

#### 7.1.1 Limited generalizability

By definition, convenience sampling chooses participants less through random or systematic processes and more depending on their ease of reach. The study’s findings might not be as applicable to a larger group. It may not be possible to generalize the results to other groups or situations because the sample may not represent the whole population.

#### 7.1.2 Sampling bias

Respondent convenience may have led to a sample not representative of population variation. Due to over- or under-representation of particular groups or traits in the study, sampling bias may occur. The external validity of the findings may be jeopardized thereby.

#### 7.1.3 Homogeneity of participants

Because the study focuses on people who engage in physical exercise for their health and well-being, it may not be as diverse as it could be regarding activity types, fitness levels, and motives. This uniformity may restrict the findings’ relevance to people who exercise for different purposes, such as managing their weight or participating in competitive sports.

#### 7.1.4 Limited exploration of other factors

Given the study’s emphasis on physical exercise for health and well-being, it’s possible that other factors that could have an impact—like pre-existing medical issues, socioeconomic position, or psychological concerns—were not considered. The study’s capacity to offer a thorough knowledge of the connection between physical activity and health outcomes may be hampered by its limited emphasis.

### 7.2 Implications of the study

#### 7.2.1 Health promotion strategies

The results highlight the need for focused health promotion initiatives that prioritize regular participation in physical activity. Public health campaigns should center on encouraging a regular exercise culture to improve general health and well-being.

#### 7.2.2 Holistic approach to health

The study emphasizes how behavior related to health, mental health, and physical exercises are all interconnected. The multidimensional nature of these linkages should be considered by intervention programs, which should take a holistic approach and acknowledge the role that physical activity plays in fostering beneficial health habits and mental well-being.

#### 7.2.3 Quality of life as a moderator

Acknowledging the moderating influence of quality of life in the association between physical activity and diverse health outcomes is imperative. The benefits of physical activity on mental health and healthy behavior may be amplified by designing interventions that improve overall quality of life.

#### 7.2.4 Individualized health plans

The significance of customized health plans is suggested by the complex and multifaceted nature of the associations found in the study. When recommending exercise programs, health practitioners should consider the patient’s quality of life and the potential moderating effects on mental health and health behavior.

#### 7.2.5 Education and awareness

Programs that raise awareness and educate people about the long-term advantages of regular physical activity are desperately needed. Educating people about the advantages of exercise for mental health, quality of life, and healthy behavior may encourage long-term participation.

### 7.3 Future scope of the study

#### 7.3.1 Longitudinal studies

Longitudinal research can shed light on the long-term advantages and possible consequences of regular physical activity by monitoring its effects on mental health, health behavior, and quality of life across time.

#### 7.3.2 Diversity in study populations

A more comprehensive range of demographics, such as age groups, socioeconomic classes, and cultural backgrounds, can provide a more thorough knowledge of the connections between physical activity, quality of life, and health outcomes.

#### 7.3.3 Exploration of moderating factors

Further research on moderating variables like social support, environmental influences, and genetic predispositions that may affect the association between physical activity and health outcomes could lead to more focused therapies and improve our understanding of the field.

## Data availability statement

The raw data supporting the conclusions of this article will be made available upon a reasonable request to the corresponding authors.

## Ethics statement

The studies involving humans were approved by Hunan City University Ethics Committee. The studies were conducted in accordance with the local legislation and institutional requirements. The participants provided their written informed consent to participate in this study.

## Author contributions

RL: Conceptualization, Data curation, Formal analysis, Investigation, Methodology, Validation, Visualization, Writing – original draft, Writing – review & editing. RM: Conceptualization, Data curation, Formal analysis, Investigation, Methodology, Software, Validation, Visualization, Writing – original draft, Writing – review & editing. ZS: Funding acquisition, Project administration, Resources, Supervision, Writing – review & editing.
